# Exercising with a Single Ventricle: Limitations and Therapies

**DOI:** 10.3390/jcdd9060167

**Published:** 2022-05-25

**Authors:** Jessica Erin Haley, Christopher Davis

**Affiliations:** Rady Children’s Hospital, University of California, San Diego, CA 92123, USA; cdavis@rchsd.org

**Keywords:** hypoplastic left heart syndrome, Fontan, exercise, cardiopulmonary exercise testing

## Abstract

Treatment for Hypoplastic Left Heart Syndrome (HLHS) and other single ventricle conditions requires a series of surgical interventions for long-term survival, typically culminating in the Fontan procedure. The result is an abnormal circulatory physiology with an absence of a sub-pulmonary ventricle. Exercise capacity in the Fontan circulation is often limited and is due to multiple factors, both central and peripheral. Multiple interventions, both pharmacologic and nonpharmacologic, have been studied to attempt to overcome these inherent limitations. This review will focus on the physiology of the exercising Fontan patient and on the interventions aimed at the enhancement of exercise capacity studied thus far.

## 1. Introduction

Long-term survival for those born with hypoplastic left heart syndrome and other single ventricle heart conditions requires either cardiac transplantation in infancy or multistage surgical palliation culminating in the Fontan procedure. This palliation, although lifesaving, creates inherently abnormal circulatory physiology which is exacerbated during the higher cardiac output needed for exercise. While exercise in Fontan physiology is well studied, due to the relatively small number of subjects most studies include multiple cardiac morphologies, and few studies are limited to HLHS patients alone. Therefore, the data presented includes all Fontan patients. The need for interventions that can improve exercise capacity in Fontan patients, including those with HLHS, is increasingly important as these patients continue to survive well into adulthood.

## 2. Fontan Circulation during Exercise

The Fontan procedure was first described in 1971 [1] and has since been utilized in patients with HLHS, as well as other types of single ventricle heart disease. It separates the systemic and pulmonary circulations by creating a total cavopulmonary connection, which excludes the sub-pulmonary ventricle [1]. In the absence of a sub-pulmonary pump, the circulation is characterized by passive pulmonary blood flow, which results in a precardiac limitation to systemic venous return and elevated central venous pressure from upstream congestion, as well as decreased cardiac output due to decreased downstream flow [2,3,4].

During exercise in a normal biventricular circulation, pulmonary artery pressure increases significantly (up to 60 mmHg, or more in athletes) which allows for the recruitment of additional lung vessels [5]. The sub-pulmonary ventricle keeps systemic venous and right atrial pressures low. In the Fontan circulation, the increase in pulmonary artery pressure during exercise results in increased venous pressures, and in a greater dependence on skeletal and respiratory muscle pumps for preload augmentation [2]. Without a sub-pulmonary ventricle, the loss of pulsatility and attenuation of high pressure, high flow pulmonary arterial circulation, which is normally seen in exercise results in less recruitment of collapsible pulmonary vessels, and thus decreased systemic ventricle preload [2]. The loss of pulsatile pulmonary blood flow over time causes an increase in pulmonary vascular resistance through vascular remodeling [6]. The filling of the systemic ventricle is also dependent on diastolic function, which is commonly abnormal in Fontan circulation, which further limits cardiac output during exercise [7]. This may be exacerbated when the systemic ventricle is the right ventricle.

## 3. Exercise Capacity in Fontan Circulation

Patients with Fontan circulation have impaired exercise capacity due to multiple factors, both central (heart and lungs) and peripheral (skeletal muscle). Each of these factors has been assessed and evaluated by various means.

### 3.1. Metabolic Assessment and Gas Exchange

It is well documented that patients with Fontan circulation have reduced peak oxygen consumption (VO_2_peak) compared to healthy controls [8,9,10,11,12], with VO_2_peak typically ranging from 60–65% predicted [4,11,13], or 21.2–27.1 mL/kg/min [14]. This decrease in VO_2_peak tends to worsen with age [9,10]. In a longitudinal multicenter study of Fontan patients, there was a decrease in %predicted VO_2_peak from 69 +/− 14% to 61 +/− 16% over a 9.4 year period [15], with a rate of decline in %predicted VO_2_peak 0.8 ± 1.7% per year [12]. This decrease in exercise capacity over time has been demonstrated to be one of the strongest predictors of adverse clinical outcomes, including mortality or transplantation [14,16], and exercise capacity below 50% predicted is the approximate threshold beyond which circulation-associated morbidities become common and symptomatic heart failure is more prevalent [17,18]. Despite the findings of decreased exercise capacity in most patients with Fontan physiology, there is significant variability amongst patients, and some have VO_2_peak values as high or higher than normal controls. These so-called “super Fontan” patients tend to be non-obese, are younger at the time of Fontan completion, report better exercise self-efficacy, and have a higher overall level of sport and physical activity participation during physical development [19].

Patients with Fontan circulation also have abnormal measures of submaximal exertion [8,13]. Measures of submaximal exertion are of particular interest in the Fontan population since it is well documented that a significant number of Fontan patients are unable to produce a maximal effort during exercise testing, as evidenced by the failure of the respiratory exchange ratio to exceed 1.0 in up to 40% of tests [14,16,20,21]. Oxygen consumption at the anerobic threshold (VO_2_@VAT) decreases with age, similarly to VO_2_peak, but typically to a lesser extent [12,13]. This relative preservation of submaximal exercise capacity is likely due to the ability to increase central venous pressure adequately to maintain ventricular preload at lower levels of exertion, while at higher levels of exertion the central venous pressures needed to maintain cardiac output exceeds physiologic limits [4].

Several submaximal exercise parameters have been used to evaluate the cardiopulmonary functional reserve, including the Oxygen Uptake Efficiency Slope (OUES) which is a measure of the efficiency of oxygen update. OUES is decreased in subjects with Fontan physiology, with a mean OUES% of 56–72% predicted, and declines with age [20,22]. Importantly, OUES is highly correlated with VO_2_peak and is independent of RER reached, making this parameter particularly useful in the Fontan population [20]. Furthermore, lower OUES has been associated with morbidity in the Fontan population, suggesting the prognostic value of this parameter [20]. The slope of the ratio of ventilation-to-carbon dioxide production (VE/VCO2 slope) during exercise is an index of ventilatory efficiency and is often abnormal in patients with Fontan circulation [14,23]. The cause of this excessive ventilation is not completely known but may be due to an increased physiologic dead space to tidal volume ratio, V/Q mismatch, or changes in the chemoreceptor setpoint for PaCO_2_ [14,23]. This is of clinical importance, as an increased VE/VCO2 slope is associated with increased mortality in patients with Fontan circulation [24].

### 3.2. Cardiac Assessment

During maximal exercise testing, patients with Fontan physiology demonstrate specific cardiovascular limitations to exercise compared to normal controls, including decreased maximal heart rate, systolic blood pressure (SBP) and O_2_ pulse [8]. These findings are consistent across various single ventricle cardiac morphologies (HLHS vs. single LV or RV) [25]. One study of young HLHS subjects (median age 6 years) who had undergone Fontan palliation, demonstrated that the mean maximal heart rate was 79 +/− 11% predicted [8]. This chronotropic insufficiency is thought to be at least in part due to underlying sinus node dysfunction, attributed to injury of the sinus node or its arterial supply, atrial dilation and cardiac hypertrophy [2]. The degree of chronotropic insufficiency typically worsens with age (peak heart rate 85% predicted in age 8–12 vs. 75% predicted in age 13–17) [10]. Age of Fontan completion may also be important. In a study of 312 patients with Fontan physiology, each year-increase in age at Fontan completion was associated with a decline of 4.1 beats/minute in heart rate reserve (defined as the difference between the maximal heart rate and resting heart rate) even after adjusting for all pertinent variables [11]. This decrease in peak heart rate and heart rate reserve has been found to be associated with poor outcomes, including death, transplantation, and hospitalizations [14].

Stroke volume is also impaired in the Fontan circulation. O_2_ pulse (VO_2_/heart rate) reflects the amount of O2 extracted per heartbeat and is a surrogate for stroke volume. Studies have shown that patients with Fontan physiology have a decreased peak O_2_ pulse compared to normal controls, in the range of 85–89 ± 22 percent predicted [8,13]. This is due to a combination of factors, including decreased systemic ventricle preload, and can be exacerbated by both systolic and diastolic ventricular dysfunction. Patients with Fontan circulation have also been found to have increased arterial stiffness compared to age-matched controls, which have been associated with lower VO_2_peak [26].

Electrocardiographic changes are seen during exercise testing in approximately 10% of patients with HLHS and Fontan circulation and include ST segment changes, varying degrees of atrioventricular block, and both atrial and ventricular ectopy [8]. Ventricular tachycardia and atrial fibrillation have been observed as well [27]. Age appears to be important, with more ECG changes noted in an older patient cohort (13–17 yo vs. 8–12 yo) [10]. The clinical significance of some of these ECG changes, particularly mild ST depression, is not fully known. In a cohort study of HLHS patients with Fontan, ST segment depression was commonly appreciated, but on further investigation, these ST segment changes were not found to be associated with coronary perfusion defects or deceased ventricular function [28].

### 3.3. Pulmonary Assessment

Many patients with Fontan circulation demonstrate abnormalities of pulmonary function. The most common finding is that of a restrictive lung disease pattern, characterized by decreased forced vital capacity. This is likely multifactorial, including having a history of multiple sternotomies, possible diaphragmatic paresis, or paralysis, and scoliosis [8]. Evidence of a restrictive ventilatory pattern is associated with lower exercise capacity in Fontan circulation [29]. Fontan patients also demonstrate reduced pulmonary diffusing capacity compared to healthy controls, which may also contribute to decreased exercise capacity [6].

### 3.4. Skeletal Muscle Assessment

There is a high prevalence of skeletal muscle and lean body mass [4,30] deficit in patients with Fontan circulation compared to age and sex-matched controls [30,31]. The causes of this include chronically elevated central venous pressure, physical inactivity, neurohormonal activation, and altered skeletal muscle blood flow [30,32]. Muscle mass deficiency is associated with impaired muscle aerobic capacity, suggesting a functional deficiency of muscle in Fontan patients [31]. Low skeletal muscle mass is correlated with lower exercise stroke volume and reduced exercise capacity [30], likely because the skeletal muscle is integral in aiding venous return to the central circulation and thus ventricular preload. Lack of lean mass and skeletal muscle also affects peripheral muscle oxygen extraction. These factors result in the musculoskeletal system being unable to meet increased metabolic demands during exercise, even if oxygen delivery remains adequate [4].

## 4. Therapeutics for Increased Fitness in Fontan Patients

There has been a limited but growing exploration of various pharmacotherapies to optimize Fontan hemodynamics during exercise. The Fontan circulation depends on passive pulmonary blood flow for cardiac output, which requires low pulmonary vascular resistance. Pulmonary arterial vasodilator medications such as phosphodiesterase 5 (PDE5) inhibitors and endothelin-1 antagonists have been studied as potential therapeutic agents. These studies demonstrate equivocal or mildly beneficial results [33,34,35,36,37]. In a study of sildenafil on exercise capacity in Fontan patients, an improvement was noted in measures of respiratory efficiency, and there was a significant increase in oxygen consumption at the anaerobic threshold in the subset of patients with elevated brain-type naturetic peptide, but there was no increase in VO2 peak observed [36]. Recently a large, multi-center, placebo-controlled study of udenafil demonstrated that six months of treatment with udenafil did not result in statistically significant improvements in oxygen consumption at peak exercise, but did demonstrate improvements in multiple measures of exercise performance at submaximal exertion (ventilatory anerobic threshold), including oxygen consumption, a ventilatory equivalent of carbon dioxide, and work rate [38].

Endothelin-1 antagonists have also been studied. In the TEMPO trial, Fontan patients treated with Bosentan for 14 weeks had modest improvement in peak oxygen consumption, exercise time, and in NYHA functional class, with few side effects reported [39].

ACE inhibitors (ACE-I) are frequently used in Fontan patients. ACE-I decreases systemic vascular resistance (SVR) and improves cardiac function and exercise capacity in adults [40,41] and children [42,43] with biventricular hearts and congestive LV dysfunction [44]. However, similar improvements in the single ventricle population have not been demonstrated. In a small, randomized, double-blind placebo-controlled trial, no change in SVR, resting cardiac index, diastolic function, or exercise capacity was found after 10 weeks of ACE-I therapy [44]. Similar findings were demonstrated in a cohort of Fontan patients with moderate-to-good systolic function, and although short-term treatment with enalapril resulted in lower resting blood pressure and pro-BNP, there was no improvement in exercise capacity, ventricular function, or arterial stiffness [45]. Side effects were common (including hypotension, syncope, dizziness, and palpitations) [45], which further questions the utility of ACE-I use for augmentation of exercise capacity in this specific population with preserved single ventricle function. This class of medication may have a more demonstrable benefit for patients with reduced ventricular function at baseline.

## 5. Nonpharmacologic Interventions

### 5.1. Regular Physical Activity and Exercise

Evidence suggests that young patients with Fontan circulation are more sedentary than healthy peers [46] and do not achieve the daily recommended levels of physical activity necessary to prevent obesity, diabetes, and atherosclerosis [46,47]. Historically, patients with Fontan circulation were discouraged from participating in competitive sports and regular physical activity due to concern for adverse events. Not only have these events during exercise been rare [48], it is now evident that regular physical activity and exercise provide a clear benefit to this patient population [19,49,50]. One study of a group of young Fontan patients found that sports practice (including leisure, competitive sports, or exercise training) was the best predictor of exercise capacity, and normal VO_2_peak was achieved in Fontan patients who reported an average of 3 h per week of sports practice [50]. Similarly, in a young adult cohort of Fontan patients, those with normal exercise capacity (high-capacity Fontan) were more likely to self-report exercise on ≥4 days per week for at least 30 min per day [49]. There was no associated increase in γGT in this high-capacity Fontan group, suggesting that an increased level of physical activity did not cause a significant increase in the degree of systemic congestion and Fontan-associated hepatic disease [49].

### 5.2. Cardiac Rehabilitation

Prescribed exercise training is an established therapy that is used routinely in individuals with various cardiopulmonary conditions [51]. Currently, exercise training is the most effective, noninvasive therapy for improving aerobic capacity in patients with Fontan circulation [32]. The benefits of exercise training include position effects on hemodynamics, both central and peripheral, as well as ventilatory and metabolic parameters. Numerous studies have demonstrated that exercise training is associated with improvement in exercise performance in patients with Fontan circulation [52,53,54,55]. All these studies demonstrate that exercise training, with programs ranging in duration from 3–12 months, results in increased oxygen consumption (range 7–16%), O2 pulse, exercise time, or workload [52,53,54,55]. These gains are more likely related to changes in skeletal muscle than to central adaptations. An evaluation of cardiac function via stress echocardiogram found that despite the increase in O2 pulse found after exercise training, there was no increase in stroke volume, suggesting that the increase in O2 pulse was due to improved efficiency of oxygen extraction by the exercising muscle rather than an increase in stroke volume [52]. Exercise training also improves measures of submaximal exertion in patients with Fontan circulation, including oxygen consumption at the anerobic threshold [52,53]. The heart rate curve, which represents the change in heart rate during various stages of exercise, also appears to decrease during submaximal levels of exercise following structured exercise training [52,55], suggesting that training causes improved cardiac output at similar submaximal heart rates. Ventilatory efficiency, as measured by VE/VCO2 slope, also improves following training. These findings of improved parameters during submaximal exercise are clinically significant, as many activities of daily living and recreational activities occur at submaximal efforts. From a respiratory standpoint, endurance exercise training is associated with improvement in forced vital capacity in Fontan patients [6]. Exercise training also improves peripheral muscle mass, muscle strength, and function in children with CHD [56]. Regular physical activity can prevent the development of Fontan-associated myopenia [57]. The increased skeletal muscle mass achieved through resistance training leads to reduced venous compliance and enhances the pump function of skeletal muscle, improving preload and ventricular filling [57]. Peak heart rate and oxygen saturations were not found to change after exercise training [52,53]. Importantly, no serious adverse events were reported during exercise training sessions, such as arrhythmias, syncope, or other symptoms other than fatigue, suggesting that this effective treatment modality is also safe [31,52]. Increased aerobic capacity is also associated with healthier end-organ (e.g., liver) function biomarkers, possibly due to chronically decreased central venous pressure, improved hemodynamics, and reduced hepatic congestion [32].

### 5.3. Pacing

Patients with Fontan physiology are often referred for pacemaker placement for sinus node dysfunction or abnormal conduction. Outside of the cases of severe chronotropic insufficiency or loss of atrioventricular synchrony, the benefits of pacing on exercise performance are limited [4]. Unlike in biventricular circulation, atrial pacing at increased rates past a certain level in the Fontan circulation does not result in increased cardiac output [58], likely due to inadequate preload to augment cardiac output at higher heart rates [5].

## 6. Summary

The Fontan circulation is the common final pathway of single ventricle palliation for patients with HLHS and other single ventricle cardiac conditions. Patients with Fontan circulation generally have impaired exercise capacity (both maximal and submaximal) due to multiple factors, both central (chronotropic insufficiency, limited preload, diastolic dysfunction, restrictive lung pattern) and peripheral (myopenia and reduced oxygen extraction). Pharmacologic interventions are of mixed efficacy. Physical activity and exercise training have been shown to be a safe and effective way to improve exercise capacity, skeletal muscle mass, and lung function. Further study into other potential means of increasing exercise capacity in a system of inherent physiologic limitations will serve this patient population well.
